# A decade of Cybathlon: impact on public visibility, scientific dissemination and technology transfer

**DOI:** 10.1186/s12984-026-01988-7

**Published:** 2026-04-16

**Authors:** Robert Riener, Luca Vassella, Peter Wolf

**Affiliations:** 1 Sensory-Motor Systems Lab, ETH Zurich, Zurich, Switzerland; 2 Spinal Cord Injury Center, University of Zurich, Zurich, Switzerland; 3 Graduate School of Engineering, Tohoku University, Sendai, Japan

## Abstract

The Cybathlon is an international competition in which individuals with physical disabilities complete activities of daily living using advanced assistive technologies, including robotic devices. International championships were held in Zurich in 2016, 2020, and 2024, with up to eight disciplines that included races using powered prosthetic limbs, exoskeletons, and wheelchairs. Between 2015 and 2024, more than 120 teams from over 30 countries participated in Cybathlon competitions and affiliated events. The aim of this work is to systematically assess the Cybathlon’s broader impact over its first decade, with particular attention to public visibility, scientific dissemination, and the translation of assistive technologies toward academic, clinical, and industrial application. To this end, we combine a longitudinal analysis of media coverage (2014–2025), a bibliometric review of scientific publications, and a survey of participating teams. A total of 6,944 media items were identified, predominantly from online sources (73.6%), with the highest coverage originating from Switzerland (26%), followed by the USA, Italy, and Germany. By January 2025, 297 scientific publications referred to the Cybathlon, with author affiliations most frequently from Switzerland, Italy, the USA, and China. Among participating teams, 79% reported accelerated prototype development due to Cybathlon participation, and over one-third attributed new educational materials, clinical methods, or collaborations to their involvement. These effects were frequently linked to early and sustained user integration, iterative testing under realistic conditions, and close interaction between engineers, clinicians, and end users. Participation directly contributed to the founding of three companies and supported five additional ventures. Additionally, at least 21 established companies participated during the various editions. Overall, the Cybathlon emerges as a unique, competition-driven innovation ecosystem that not only increase public awareness of assistive technologies but also shapes research priorities, support user-centered design practices, and facilitates pathways toward clinical adaptation and commercialization.

**Supplementary Information:**

The online version contains supplementary material available at 10.1186/s12984-026-01988-7.

## Introduction

### Addressing the gaps within assistive technologies

In 2022, around 1.3 billion people were living with some form of disability, a number expected to rise as the global population ages [28]. Nowadays, approximately 80 million people have relied on wheelchairs, and over 57 million have experienced limb amputations (). Unfortunately, current assistive technologies often do not offer usability and functionality that would be necessary to support individuals in their everyday lives. For instance, most wheelchairs cannot climb stairs, arm prostheses do not enable versatile hand functions, and many orthotic and prosthetic devices have limited power supply. These limitations can be due to a lack of communication between developers, people with disabilities, therapists, and clinicians, which leads to a disregard of user needs and requirements. Other reasons could be that the health status, level of lesion, or financial situation of potential users are so severe that they are unable to use the technologies, if they are available at all. Furthermore, physical but also attitudinal barriers in public environments make the use of assistive technologies often very cumbersome or even impossible.

Consequently, there is a need to further push the development of assistive devices by pooling the efforts of engineers and clinicians to improve technologies, together with the feedback and experiences of the users of the technologies, in order to provide functionality and usefulness and, thus, enhance the quality of life for people with disabilities. In response to these challenges, ETH Zurich launched the Cybathlon, a new kind of platform with the aim of promoting the development of useful assistive technologies by organizing international championships and other events [13, 16, 23, 24].

### The Cybathlon editions

In contrast with the Paralympics, where parathletes aim to achieve maximum performance, at the Cybathlon championships, people with physical disabilities, such as limb paralysis or limb amputations, compete against each other at tasks of daily life, with the aid of advanced assistive devices including robotic technologies. Six disciplines were part of the first two editions that took place at the SWISS Arena in Kloten close to Zurich in October 2016, and in a remotely broadcasted global form with a central coordinating hub at ETH Zurich in November 2020.

The six disciplines comprised races with powered leg prostheses, powered arm prostheses, functional electrical stimulation (FES) driven bikes, powered wheelchairs and powered exoskeletons. The sixth discipline was a racing game with virtual avatars that are controlled by brain-computer interfaces (BCI). At the Cybathlon 2024, two more disciplines were added, namely, a vision assistance race for people with severe seeing impairments helping them to navigate through a home and public environment, and an assistance robot race that helps people with severe motor impairments to interact with their physical environment.

The functional and assistive devices used were prototypes developed by research labs or companies, or commercially available products. The competitors are called pilots, as they must control a device that enhances their mobility, such as a pilot controlling a plane or racing car. The teams each consist of a pilot together with scientists and technology providers, making the Cybathlon also a competition between companies and research laboratories. More information can be found on the Cybathlon website https://cybathlon.com/en.

### Aims of this work

Between 2015 and 2024, the Cybathlon hosted multiple competitions and affiliated events involving more than 120 teams from over 30 countries. This study elaborates on the impact of these activities, with particular attention to public visibility on different media, scientific dissemination, and the transfer of technology to industry. It included ongoing and completed product developments as well as progress toward commercialization. Data sources comprised media coverage from 2014 to 2025, academic dissemination indexed in databases such as Google Scholar, and responses to a questionnaire distributed to all participating teams.

##  Methods

### Media coverage data collection and analysis

Over the years, media coverage has played a critical role in disseminating Cybathlon values, outcomes, and global visibility. It has further contributed to attracting sponsoring partners, donors, participating teams, and audiences, both in the stadium and via online platforms.

Media coverage data of the Cybathlon were collected between January 2014 and May 2025, following a structured, multi-source approach (Table 1). The process combined institutional media monitoring, organizational archives, and targeted digital and social media searches to ensure comprehensive coverage across the DACH region (i.e., Germany, Austria, Switzerland) and beyond. To supplement search engine results, several contributors undertook manual hand-searches (category “Manual” in Table 1) by directly reviewing magazines, newspapers, and websites to identify and compile Cybathlon-related articles that search engines may have missed.

**Table 1:** Main data sources, extraction periods, extraction methods and further information to the data sources

Source/tool	Extraction period	Extraction method	Description and role in data collection
Muck Rack^1,2^	2019–2025	Software	Media extract provided by ETH Corporate Communications to search for “Cybathlon” in news outlets and magazines across the DACH region and internationally.
ARGUS DATA INSIGHTS^3,4^	2014–2024	Software	Media extract provided by ETH Corporate Communications to identify articles, broadcasts, and online mentions related to Cybathlon across the DACH region and internationally.
Cybathlon Organization	2014–2025	Manual	Media materials and press documentation (journals, websites, magazines, etc.) retrieved through hand-searches by the Cybathlon organizing team.
Magazines & Journals	2014–2025	Manual	Additional magazine and journals articles collected over the years by the Sensory-Motor Systems Lab at ETH Zürich.
Manual Searches	2014–2025	Manual	Hand-search performed by Cybathlon staff and the authors of this article to complement the data obtained from automated and platform-based extractions.
YouTube^5^	2014–2025	Software	Data extracted using a Python script and the Google Cloud API, searching for “Cybathlon” and its translations in multiple languages to ensure global coverage.
Apple Podcasts^6^	2014–2025	Manual	Podcasts found on the Apple Podcasts platform through hand-searches using the keyword “Cybathlon”.
X (Twitter)^7^	2014–2025	Software	Data collected using Tweet Binder, quantifying tweets and reposts related to Cybathlon.

^1^ Media monitoring tool from Muck Rack, LLC, 2025 (https://muckrack.com/)

^2^ Muck Rack data extraction: 1st November 2019 to 6th March 2025

^3^ Media monitoring tool from ARGUS DATA INSIGHTS Schweiz AG, 2024 (https://www.argusdatainsights.ch/en/)

^4^ ARGUS data extraction: 1st January 2014 to 31st December 2024

^5^ YouTube data extraction: 1st January 2014 to 5th May 2025

^6^ Apple Podcasts data extraction: 1st January 2014 to 1st April 2025

^7^ X (Twitter) data extraction: 1st January 2014 to 26th May 2025

For YouTube, a Python script (Python Software Foundation, [21]) was implemented on Visual Studio Code to search for videos containing the term “Cybathlon” or its translations into Japanese, Hindi, Russian, Korean, and Chinese within the video title or description. Translated search terms were limited to languages spoken in countries with Cybathlon team participation and demonstrable media coverage, based on preliminary searches. The audiovisual content and transcripts were not analyzed. The script was executed separately for each year from 2014 to 2025 and searched both titles and descriptions for exact keyword matches. The complete list of keywords included:

- English: “Cybathlon 2016”, “Cybathlon 2020”, “Cybathlon 2024”, “Cybathlon”.
- Japanese: “サイバスロン 2016”, “サイバスロン大会 2016”, “サイバスロン 2020”, “サイバスロン大会 2020”, “サイバスロン 2024”, “サイバスロン大会 2024”.
- Hindi: “साइबाथलॉन 2016”, “साइबैथलॉन 2016”, “साइबाथलॉन 2020”, “साइबैथलॉन 2020”, “साइबाथलॉन 2024”, “साइबैथलॉन 2024”.
- Russian: “Кибатлон 2016”, “Кибатлон 2020”, “Кибатлон 2024”.
- Korean: “사이배슬론 2016”, “사이배슬론 2020”, “사이배슬론 2024”.
- Chinese: “机械奥运会 2016”, “机械奥运会 2020”, “机械奥运会 2024”.

In English, the keyword search was structured by the Cybathlon edition year. The script first identified videos containing the term “Cybathlon” in the title or description in combination with a specific edition year (2016, 2020, 2024), followed by searches using the term “Cybathlon” alone. For Japanese, Hindi, Russian, Korean, and Chinese, the translated term “Cybathlon” was searched in combination with the edition year to enhance accuracy and relevance. To prevent duplicate counts that could bias the results, videos with identical URLs were included only once.

For X (formerly Twitter), data were extracted using a commercial social media analytics tool. A subscription to the “Starter” plan of Tweet Binder (Tweet Binder, [26]) provided historical tweet counts referencing “Cybathlon”, using the keyword “Cybathlon”.

LinkedIn was not included in the longitudinal analysis because historical post and hashtag data beyond approximately 12 months are not accessible via the platform interface or official APIs, precluding a reproducible analysis over the full 2015–2025 study period.

All data, except for that from X (Twitter), were consolidated into a centralized Excel file. The file was processed through a data-cleaning procedure, including the treatment of missing data, resolution of format inconsistencies and typographical errors, and the removal of duplicates [8], resulting in a final dataset of 6’944 single reports. For X (Twitter), historical tweet count reports were generated directly using Tweet Binder, an X (Twitter) hashtag analytics tool owned by Audiense Limited (https://www.tweetbinder.com).

Descriptive statistics and data visualization [14] were performed on the dataset. Tables and histograms were created in Excel to summarize and visualize the distribution of coverage volumes by year, media type, and geographical region. Additionally, Python (Python Software Foundation, [21]) was used to generate a world map illustrating the countries represented in the dataset. To better understand the global spread of Cybathlon, the presence of the word “Cybathlon” in titles was also evaluated as an indicator of direct visibility.

No inferential statistical tests (e.g., p-values or hypothesis testing) were performed, as the aim was not to test hypotheses or generalize findings, but rather to provide a comprehensive overview of the available data.

### Scientific impact data collection and analysis

Since its inception, Cybathlon has served not only as a platform for showcasing advanced assistive technologies but also as a catalyst for scientific research across multiple disciplines. Its unique format, bringing together researchers, clinicians, engineers, students, and individuals with disabilities, has fostered a collaborative environment that continues to support the development and dissemination of new knowledge [16, 23].

A meaningful indicator of scientific impact is the body of publications that reference Cybathlon in any capacity. These works span diverse topics, including the design and evaluation of assistive devices, user experience studies, the development of novel rehabilitation approaches and clinical therapies, and the broader societal impact of technology on people with disabilities. Tracking and analyzing these publications provides insight into Cybathlon’s contributions to the scientific community over the past decade.

To document the scientific output related to Cybathlon, both manual and software-assisted searches were conducted to collect all publications mentioning the term “Cybathlon” up to 17 January 2025. The official Cybathlon website provided a baseline set of relevant publications, including those authored by participating teams. Additional publications were identified through systematic searches of PubMed, ScienceDirect, and Scopus. Where available, publication lists were exported directly as CSV files. Furthermore, the software “Publish or Perish” (A.W. Harzing, [3] was employed to extract supplementary publication data from PubMed, Scopus, CrossRef, and Google Scholar.

After the compilation of all publications up to 17 January 2025, the dataset was manually updated in May 2025 by adding further publications reported by previously participating teams through a survey.

As a comparative analysis, searches were conducted on four academic platforms including Google Scholar, Scopus, PubMed, and ScienceDirect, using the keyword “Cybathlon”. For each platform, the total number of search results was recorded. Where possible, results were further restricted to scientific publications: on Google Scholar, the “Review articles” filter was applied, and on Scopus, results were limited to “Article” and “Conference Paper.” All searches were restricted to publications appearing between 1 January 2014 and 26 May 2025. This approach enabled a comparative overview of the representation of Cybathlon-related publications across major scientific databases.

All identified publications were consolidated into a centralized Excel file. The dataset was refined by standardizing and correcting author names, adding the journal’s country of origin and authors’ affiliation countries, updating other bibliographic details, and removing duplicate entries. The country of origin for each publication was coded using the three-letter ISO 3166 alpha-3 standard, as listed on the IBAN country codes page (IBAN, [11]).

After refinement, the final dataset contained 297 scientific publications that included the keyword “Cybathlon”. To assess the relevance of each publication, entries were categorized into three groups based on the location of the keyword: (i) in the title (regardless of whether it also appeared in the abstract or body), (ii) in the abstract but not the title (with possible mentions in the body), or (iii) exclusively in the body text. Publications were assigned hierarchically: priority was given to the title, followed by the abstract, and finally the body.

Descriptive statistical analyses were conducted to summarize the dataset. Tables, histograms, and pie charts were created in Excel to visualize the distribution of publications by discipline, source type, and other relevant categories. Consistent with the descriptive aims of this study, no inferential statistical analyses (e.g., p-values or hypothesis testing) were performed. Instead, the analysis was limited to summarizing and illustrating dataset characteristics without making claims about statistical significance or broader generalizations.

### Survey with teams

A survey was conducted with teams that had participated in one or more of the Cybathlon editions held in 2016, 2020, and 2024. The primary objective was to assess the event’s impact from the perspective of the participating teams. Specifically, the survey examined how Cybathlon contributed to or accelerated knowledge transfer into scientific publications and other dissemination channels, facilitated the development of new therapies or rehabilitation methods, advanced product development and market accessibility, increased public visibility, and fostered collaboration among stakeholders.

The survey was developed using Microsoft Forms (Microsoft Corporation, 2024) and comprised 62 questions tailored to address the different roles within each team. The main section was directed toward team managers and members, while pilots were asked to complete a shorter, dedicated section. Branching logic ensured that respondents were presented only with questions relevant to their role and prior answers. Consequently, although the full survey contained 62 items, most teams answered between 20 and 30 questions, depending on their experience and response pathways.

The questionnaire included both closed-ended and open-ended questions to capture quantitative data and qualitative feedback. The initial section gathered general team information, while the final section provided space for open feedback and the option to share contact information for potential follow-up. The questionnaire was administered in English. Ethical approval for the study was obtained from the local ethics committee of ETH Zurich (Project 24 ETHICS-398) prior to distribution. The questionnaire is provided in the Appendix (see Additional file 2).

The survey was distributed electronically to the team managers of all groups that participated in the main Cybathlon editions of 2016, 2020, and 2024. Invitations were sent on 18 December 2024, with follow-up reminders issued between January and April 2025. In total, 122 teams were invited. A total of 88 fully completed questionnaires were received: 67 from team managers, 13 from team members, and 8 from pilots. The average time taken to complete the entire questionnaire was 55 min. Among the 80 answers recorded from team managers and members, some responders represented more than one team, and in other instances multiple representatives from the same team completed the survey. After reviewing the responses, we were able to identify 76 different teams represented in the survey at least once, corresponding to a response rate of 62.3% among the 122 invited teams. Since multiple responses could originate from the same team and some respondents were members of more than one team, results are reported at the respondent level (i.e., 80 team managers or members) rather than the team level.

A mixed-methods approach was applied to analyze the submitted questionnaires. Survey responses were exported and analyzed using Python (Python Software Foundation, [21]). Quantitative data from closed-ended questions were summarized using descriptive statistics, while qualitative responses were thematically grouped to identify recurring themes.

Pilot participation was limited: only 8 pilots completed the pilot-specific section, with one team represented twice, yielding an approximate pilot response rate of 5.6%. Due to this low response rate, pilot-focused analyses and results were not included in this study.

For clarity and ease of interpretation, the original survey question labels were shortened and simplified for data analysis. These concise labels are used throughout the results section and in the accompanying graphs. The renaming of the original questions for presentation purposes is described in Appendix (see Additional file 1).

## Results

### Media coverage and event attendance

In total, 6’944 media items related to Cybathlon were collected, with the majority, 73.6%, originating from web-based sources. Other content types include YouTube (12.1%), print (7.4%), while podcasts, TV, and radio each accounted for about 1% or less of the total coverage (Table 2). Some items were found that could not be clearly assigned to print or web (3.6%).

**Table 2:** Count of media items according to different sources

Type of source	Single reports count	%
Webpages	5’114	73.6
YouTube	838	12.1
Print	516	7.4
Print/Web^8^	247	3.6
Podcasts	96	1.4
TV	81	1.2
Radio	52	0.7
Total	6’944	100

^8^ Sources for which it was not possible to clearly determine whether they were print or online

Of the 6’944 media items analyzed, 2’163 (31.1%) included the term “Cybathlon” in the title, while the remainder did not. This suggests that at least nearly one-third of the coverage explicitly focused on the event rather than mentioning it only incidentally.

**Figure:**
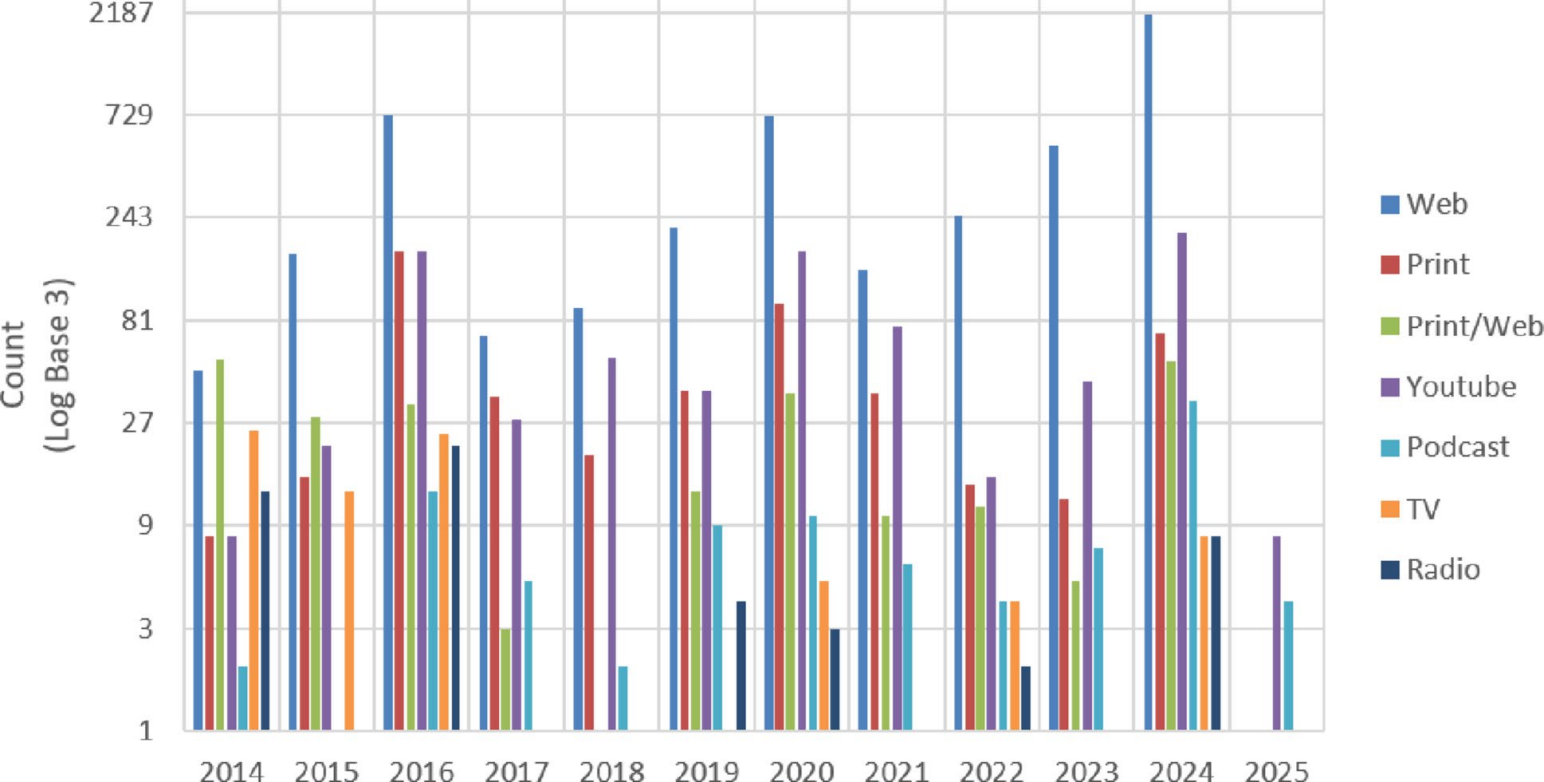
**Fig. 1** Coverage of cybathlon in various media in different years. Note that numbers are shown logarithmically to the log base of 3

Overall, media coverage increased over time with pronounced peaks in the years of the Cybathlon main editions (2016, 2020, and 2024, as presented in Fig. 1).

**Fig. 2:**
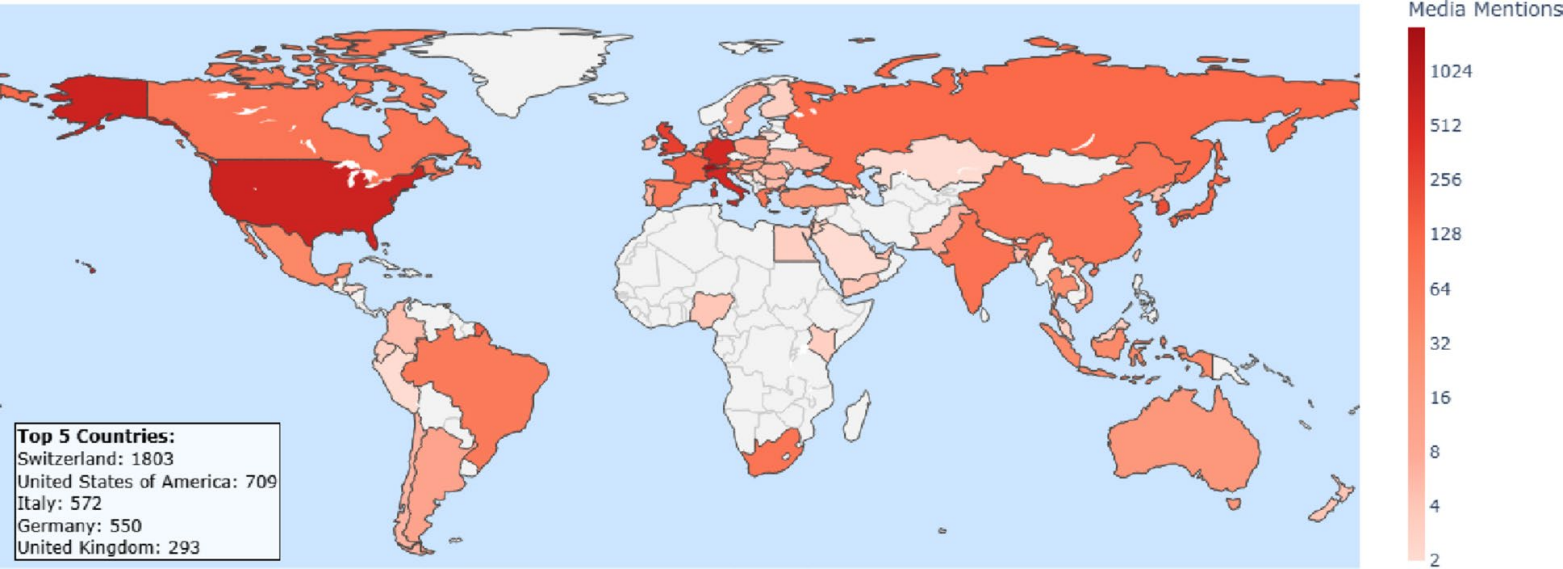
Media coverage around the World between 2014–2025. The different shades of red express the intensity of media coverage in different countries

Media mentions, classified by the country of origin of the source, were recorded across all continents. The highest proportion was observed in Switzerland, accounting for 1’803 of 6’944 items (26%), followed by the United States (10.2%), Italy (8.2%), Germany (7.9%), and the United Kingdom (4.2%) taking the publication years 2014–2025 (Fig. 2). Continents with the highest media coverage were Europe and North America. Localization was conducted using media items from print, web, television, and radio sources. Items from platforms such as YouTube, podcasts, and X (Twitter) were excluded from the mapping due to challenges in reliably determining their geographic origin.

**Fig. 3:**
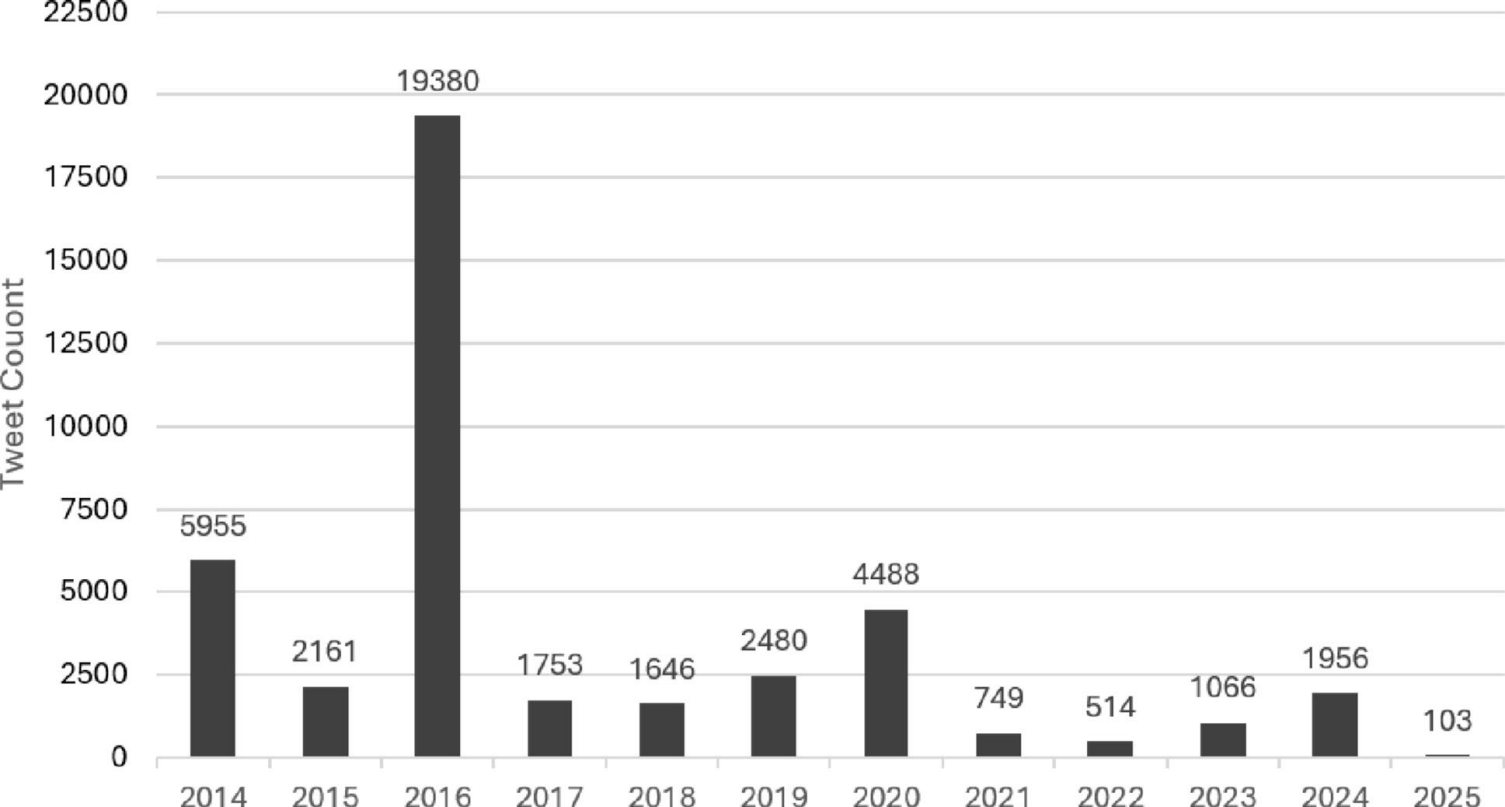
Count of Cybathlon tweets on platform X (Twitter)

A total of 42’251 tweets and reposts about Cybathlon were made between 2014 and 2025 (Fig. 3). The first edition of the event in 2016 accounted for 45.9% of all tweets during this period. The pronounced spike coincides with the inaugural Cybathlon and a period when Twitter played a particularly prominent role in live event- and news-oriented communication. Smaller but clearly discernible peaks are also visible in the years of subsequent Cybathlon championships, indicating that social media engagement responds strongly to the event itself. Lower activity in later editions likely reflects both reduced novelty and a broader shift toward diversified media platforms, rather than diminished overall visibility [2, 7].

In addition to media coverage, Cybathlon attracted substantial public attendance and online viewership at its main events. In 2016, the competition was held exclusively at the SWISS Arena, with over 5,000 spectators on site and approximately 4,700 live viewers of the Swiss television broadcast. Due to the COVID-19 pandemic, the 2020 edition was organized across 32 decentralized hubs, reaching around 13,400 spectators via live online streams. In 2024, Cybathlon returned to the SWISS Arena, complemented by seven international hubs, attracting more than 6,000 spectators on site over three days and over 21,000 livestream viewers. All figures are based on internal final event reports.

### Scientific impact

**Fig. 4:**
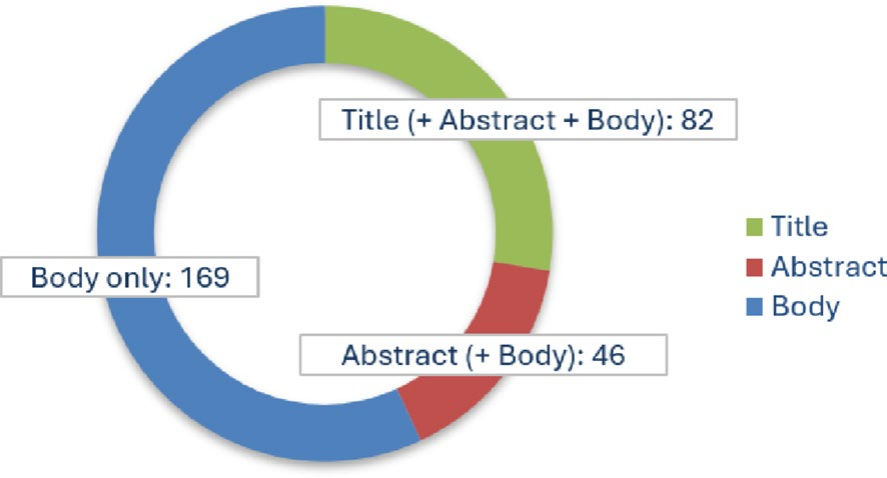
Number of scientific publications containing the term “Cybathlon” in the title, abstract and/or text body. Total number of publications found was 297

Of the 297 scientific publications identified, 82 (28%) included the term “Cybathlon” in the title, 46 (15%) mentioned it in the abstract but not in the title, and the remaining 169 (57%) referenced the term only in the main text (Fig. 4).

**Fig. 5:**
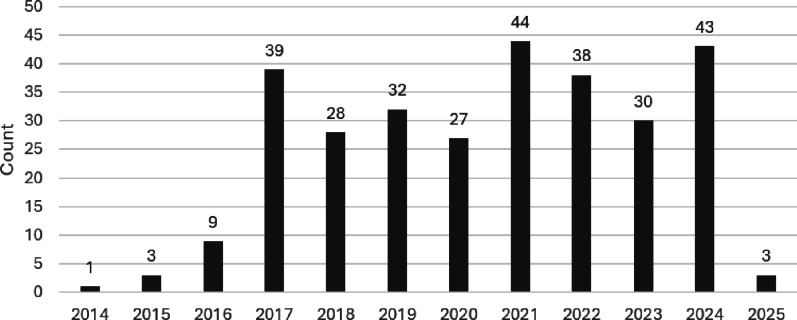
Number of scientific publications containing the term “Cybathlon” sorted by year. The search was conducted until May 2025

The first Cybathlon-related publications appeared in 2014. The annual number of publications increased progressively until 2017 (Fig. 5). From 2018 onward, the yearly output ranged between 27 and 44 publications, with peaks in 2021 (44) and 2024 (43).

**Fig. 6:**
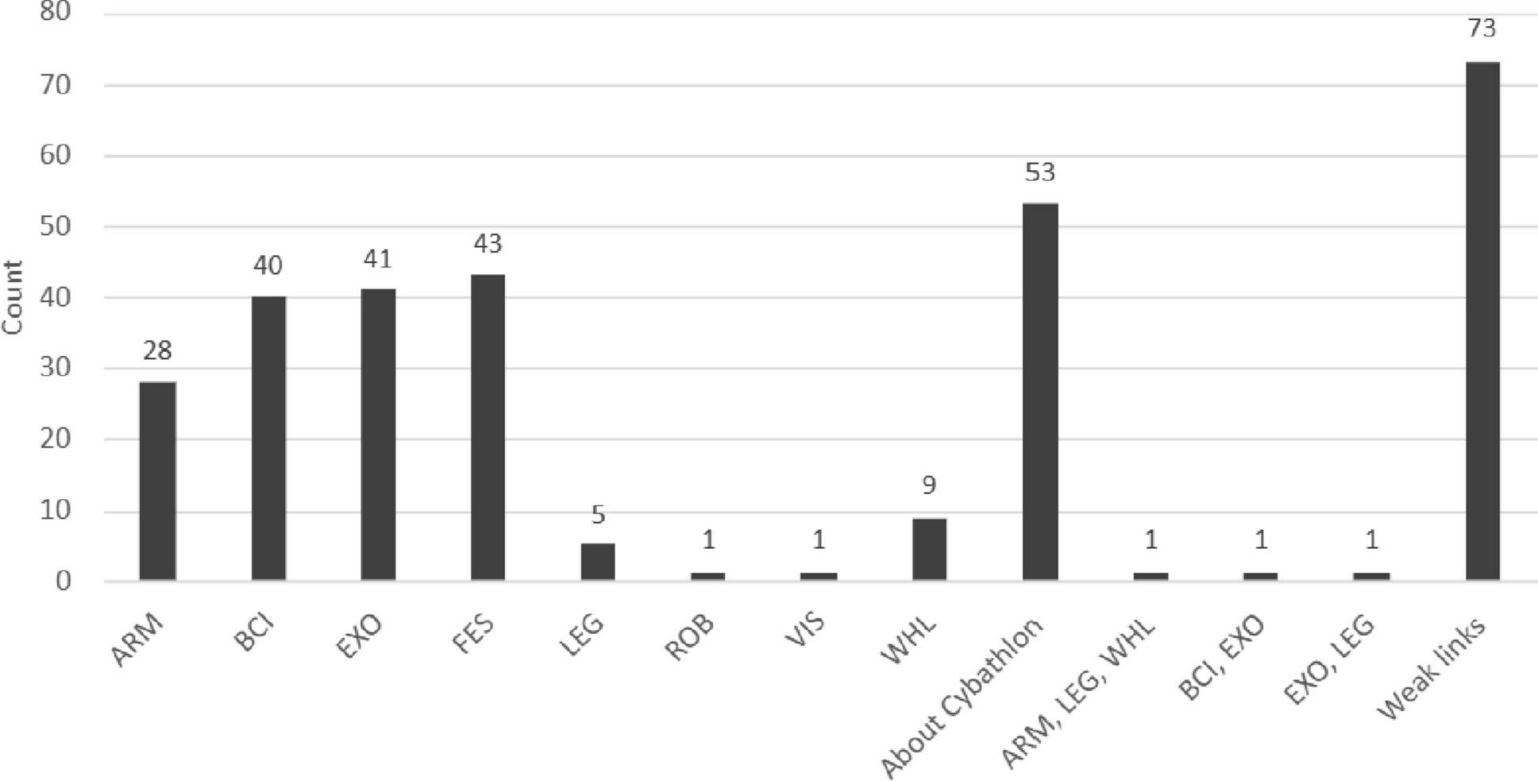
Scientific publications mentioning the term “Cybathlon” sorted by discipline, namely Arm Prothesis Race (ARM), Brain-Computer Interface Race (BCI), Exoskeleton Race (EXO), Functional Electrical Stimulation Bike Race (FES), Leg Prothesis Race (LEG), Assistance Robot Race (ROB), Vision Assistance Race (VIS), Wheelchair Race (WHL). The term “About Cybathlon” means that the publication did not focus on a specific discipline, but on the event or the technology in general. “Weak links” denotes publications that mention Cybathlon only marginally in the text body, without a substantive thematic link

The distribution of scientific publications by Cybathlon discipline indicates that the majority did not focus on a single discipline or a specific subset. Among discipline-specific publications, the highest number addressed the Functional Electrical Stimulation Bike Race (FES), followed by the Exoskeleton Race (EXO) and the Brain–Computer Interface Race (BCI). Other disciplines, including the Arm Prothesis Race (ARM), the Leg Prothesis Race (LEG), and the Wheelchair Race (WHL), were represented less frequently (see Fig. 6).

In terms of publication type, most Cybathlon-related outputs were peer-reviewed journal articles (*n* = 192, 64.6%), followed by conference papers (*n* = 45, 15.2%) and book chapters (*n* = 26, 8.8%). Additional types included general journal contributions (e.g., focus articles; *n* = 21, 7.1%) and academic theses (bachelor’s and master’s; *n* = 6, 2.0%). Seven of the 297 publications could not be clearly classified or were still under review at the time of analysis.

Beyond publication counts and temporal trends, the identified 297 Cybathlon-related publications were classified according to the type of knowledge generated. The majority of publications are of a technical nature (227 papers, 76.4%), primarily published in engineering journals or conference proceedings. Within this group, 93 publications (31.3% of all papers) are review, survey, or general overview articles (partly mentioning Cybathlon only marginally), while 8 publications (2.7%) specifically address usability, user needs, or user-centered design aspects.

A further 35 publications (11.8%) are of a more general nature, focusing on the Cybathlon event itself or drawing comparisons to other competitive or challenge-based formats such as the Olympic and Paralympic Games or DARPA challenges. Clinical and therapeutic aspects, including case studies, are addressed in 19 publications (6.4%), predominantly published in non-technical outlets. Societal, ethical, or educational perspectives are discussed in 12 publications (4.0%). The remaining 4 publications mention the Cybathlon without a substantive thematic focus.

**Fig. 7:**
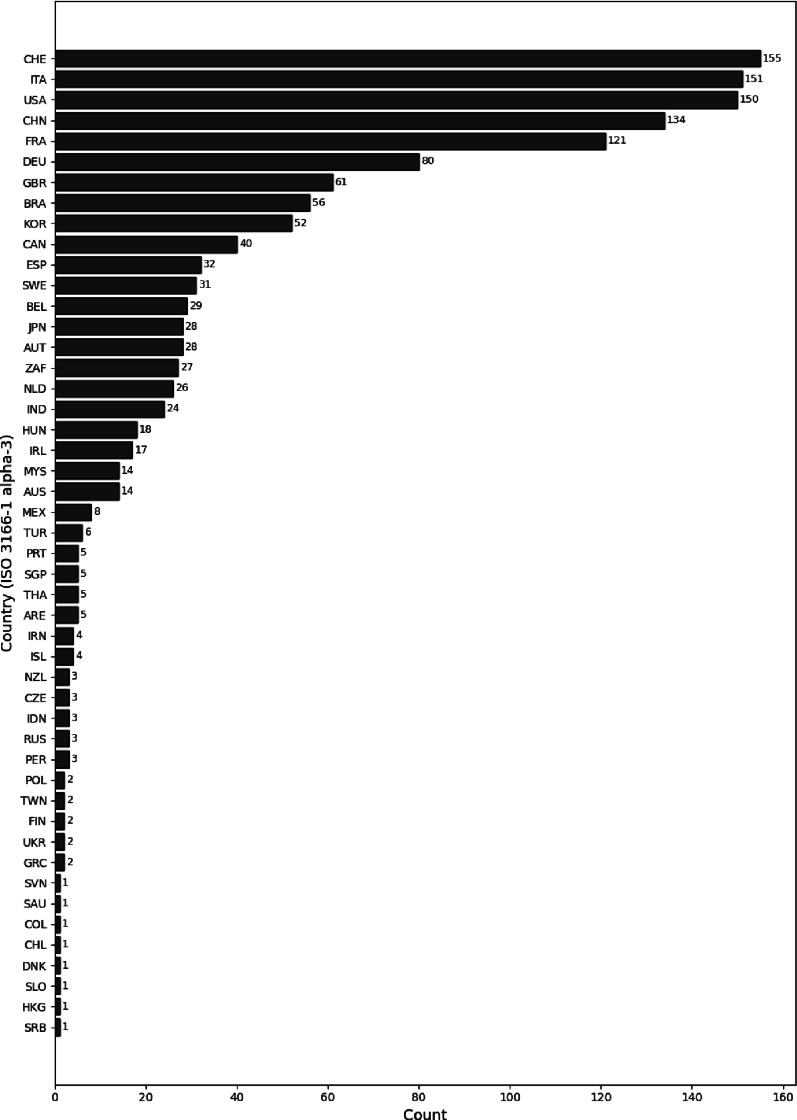
Number of scientific publications sorted by authors’ country of affiliation

One method to determine the engagement of countries involves analyzing the affiliations of authors listed in relevant publications and counting the corresponding countries of these affiliations (see Fig. 7). In total, 1’363 author affiliations were recorded and categorized by country. Authors who contributed to multiple publications were counted multiple times. The highest numbers of author affiliations originated from Switzerland (11.4%), Italy (11.1%), and the USA (11.0%), followed by China (9.8%) and France (8.9%).

### Experience of developers

**Fig. 8:**
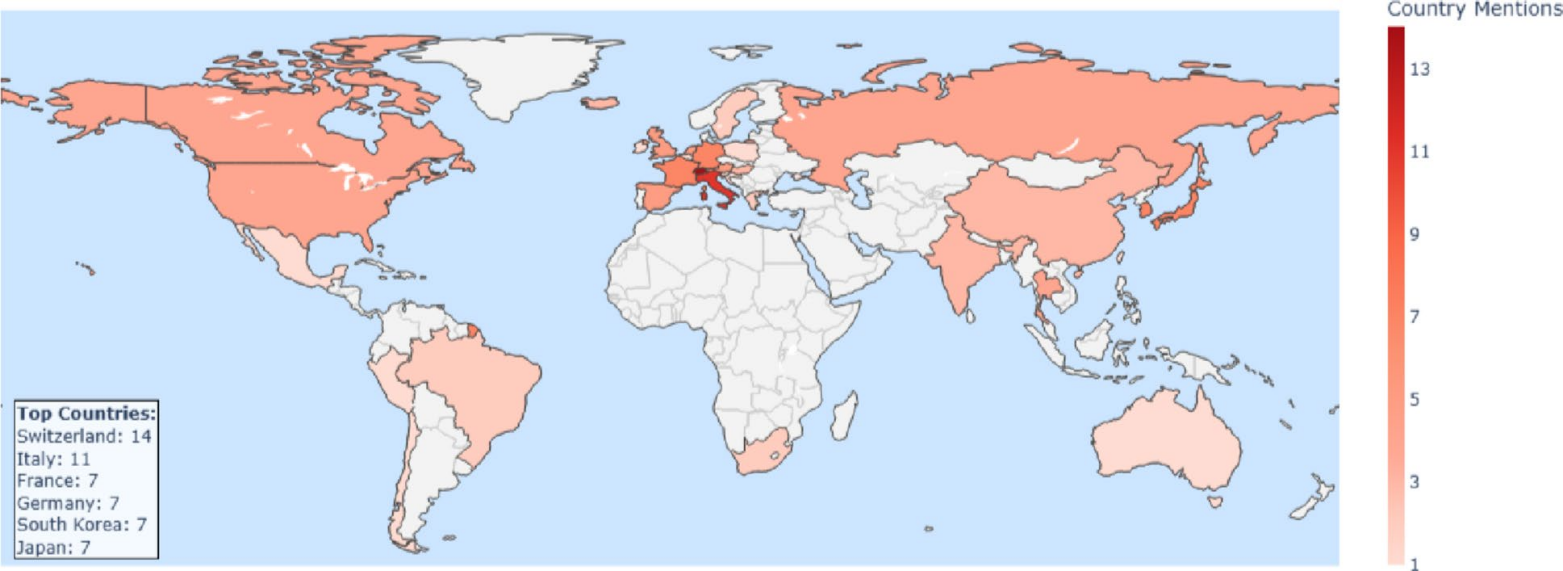
Geographical distribution of teams participating in the Cybathlon (2016, 2020, 2024)

Since its first edition in 2016, Cybathlon has attracted teams from all around the world to showcase their knowledge and developments in assistive technologies, ranging from Europe and the Americas to Asia, and Australia; in total 33 countries were involved in all three editions of the championship (Fig. 8).

According to the survey performed in this study, 59 (73.8%) of the team managers and members who filled out the questionnaire indicated that Cybathlon had a considerable or extreme impact on their team’s reputation, while 17 (21.3%) reported only a minor impact, and 4 (5.0%) reported no impact at all. More specifically, regarding the impact on media presence, the following responses were given: Sixty-one responders (76.3%) recognized the Cybathlon as the main driving or facilitating force for media recognition, including being featured or invited to various platforms. Fifty-eight responders (72.5%) say that their participation in the Cybathlon is the main reason or facilitating force that has attributed most of their popularity to social media.

The responses referring to research and education, 40 team managers and members (50.0%) reported that their participation with a team at the Cybathlon led or facilitated the release of scientific publications about their assistive device developments. And 29 team managers or team members (36.3%) indicated that their participation led or facilitated the development of new educational materials, such as curricula or lectures.

Referring to the clinical context, 28 team managers and team members (35.0%) said that their participation in the Cybathlon did lead or facilitate the development of novel rehabilitation methods or therapies. And 26 team managers and members (32.5%) indicated that their participation led or facilitated the establishment of collaborations with clinical sites, such as therapy centers. Eleven responders (13.8%) reported that their involvement with NGOs and similar organizations was a direct result of or facilitated due to their participation in the Cybathlon.

A total of 63 team managers and members (78.8%) stated that Cybathlon pushed the development of their prototype. Regarding technology transfers, 46 (57.5%) team managers and members perceived a considerable or extreme impact, whereas 22 (27.5%) reported only a slight impact, and 12 (15.0%) indicated no impact. Twelve team managers and members (15.0%) reported that their device is currently available on the market after being showcased during any of the last three Cybathlon editions.

The participation at Cybathlon was the main reason for the founding of the three companies TWIICE SA, Scewo AG, and SensorStim Neurotechnology GmbH. And it facilitated the founding of five other companies (Kurage SAS/SASU, Haptika Medical Technologies SAS/SASU, Circleg AG, Kindness Robot at Taipei Medical University, and MYLEG AG). Additionally, at least 21 already established companies and startups participated in the competitions during the various editions. Some of them, such as Ottobock GmbH, Össur h.f., Katervil LLC, or Angel Robotics Co. Ltd, used the Cybathlon to release new product versions or functions. Others, such as ReWalk Robotics Ltd., HASMOMED GmbH, Xiborg co. ltd., B-free Technology Ltd., or Wandercraft SAS took the opportunity to showcase and advertise their products.

In response to the question “Did your team receive any funding for the development of the product you brought to the Cybathlon?”, 61 team managers and members (76.3%) reported that they were receiving fundings for their device development, while 19 (23.8%) did not receive fundings.

As for “During your journey to Cybathlon, did your team engage in any crowdfunding campaigns for your product?,” only 7 team managers and members (8.8%) reported participating in crowdfunding initiatives, whereas 73 (91.3%) did not.

Among the responding team managers and members, 44 (55.0%) of them plan future scientific publications, 23 (28.8%) intend to develop new educational materials, 36 (45.0%) anticipate contributing to new rehabilitation methods or therapies, 55 (68.8%) foresee collaborations with clinics or therapy centers, 24 (30.0)% are considering founding startups or spin-offs, 33 (41.3%) expect to bring products to market, and 22 (27.5)% plan to engage with NGOs or similar organizations in the future.

**Fig. 9:**
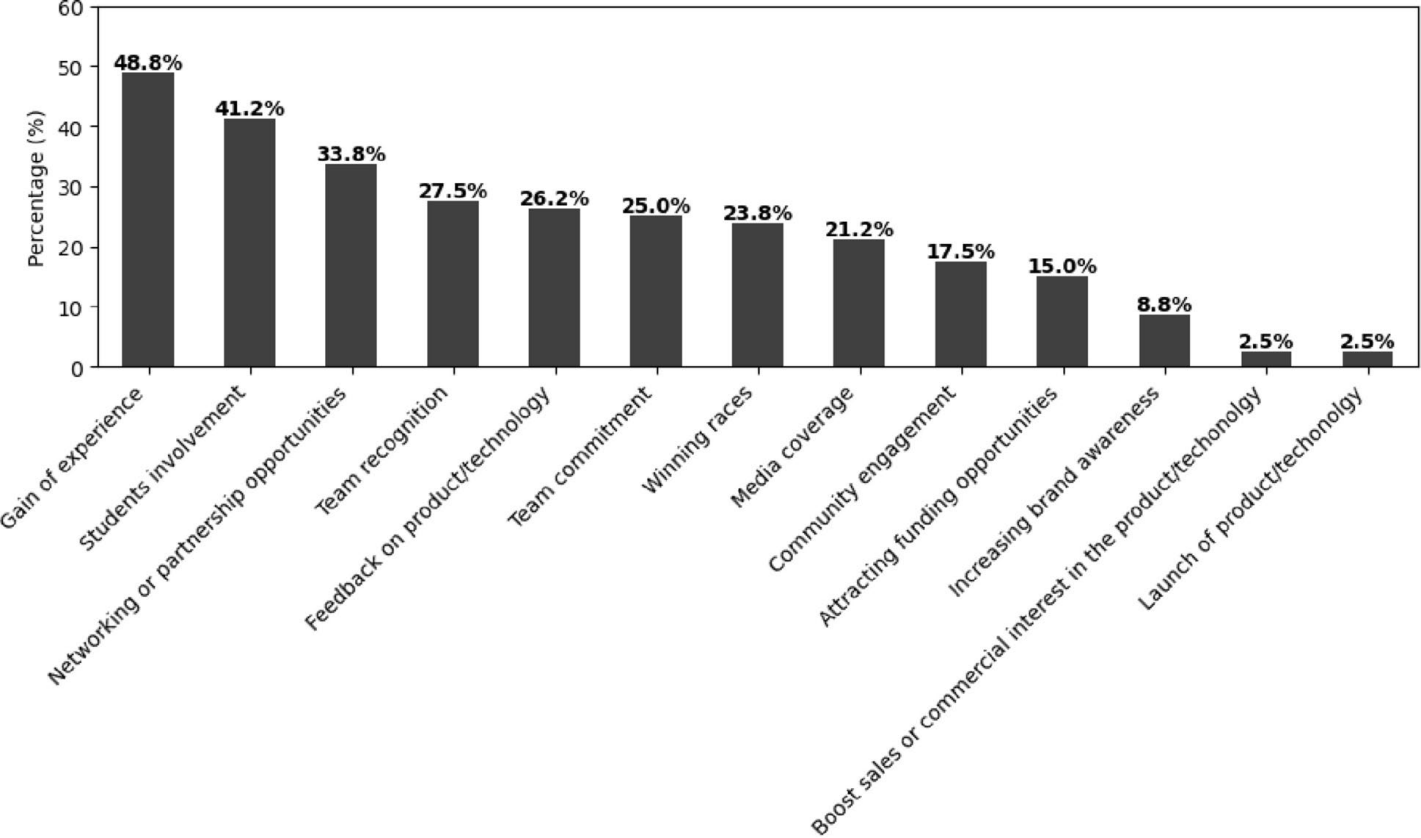
Reasons for participating in the Cybathlon from the teams’ perspective. Percentages are based on 80 respondents. Respondents could select up to three aspects in answer to the question “What aspects make participating in the Cybathlon worth the journey for you and your team?”

Note that the survey responses reflect self-reported perceptions of impact on device development, evaluation practices, and collaboration, and do not allow causal attribution of specific technological or clinical advances.

The survey results indicate that nearly half of the team managers and members identified gaining experience as a key benefit of participating in the Cybathlon (Fig. 9). Student involvement and opportunities for networking or partnership were also highly valued, each selected by over a third of the respondents. Several other aspects, such as team recognition, feedback on product or technology, and team commitment, were also frequently mentioned, reflecting a broad appreciation for both the developmental and collaborative opportunities facilitated by the event.

## Discussion

### Media coverage

#### Interpretation of media coverage results

The media analysis of Cybathlon from 2014 to 2025 demonstrates a substantial expansion of its global presence. Coverage exhibited distinct peaks in the years of the main events (2016, 2020, and 2024), reflecting the competition’s ability to attract public and journalistic attention during these periods. This temporal pattern is consistent with other major sporting events, such as the Paralympic Games, where media interest intensifies competition dates and subsequently declines [10]. Media coverage was predominantly web-based, followed by video platforms such as YouTube, reflecting a broader shift away from traditional media toward digital channels that enable faster dissemination and global reach [6, 25]. In response to these trends, the Cybathlon format has evolved over time, combining centralized events with distributed and digital components to expand accessibility and visibility.

A geographical analysis of media mentions indicates that Cybathlon achieved coverage across nearly all continents, with the highest intensity observed in Europe and North America. Switzerland accounted for the largest share, which is expected given that the main Cybathlon championships were hosted there. In contrast, low- and middle-income countries in regions such as Africa, parts of Asia, and Latin America were markedly underrepresented. This underrepresentation may be attributed to several factors, including the limited participation of teams from these regions, restricted internet access, language barriers, and lower levels of public and media interest in science, technology, and disability sports. Similar disparities in media attention have been observed in other international events (International Telecommunication Union (ITU), [12, 18]).

#### Narratives

A closer examination of Cybathlon media coverage reveals recurring patterns in both content and framing. Many articles adopt a medicalized narrative, emphasizing the overcoming of disability through technology, and frequently use terms such as “severe disabilities,” “bionic Olympics,” and “superhuman.” While some of these articles focus on the compensation of physiological impairments, risking the reinforcement of a deficit-based view of disability and perpetuating the “supercrip” stereotype; others shift the focus toward glorifying technological innovation. In these cases, assistive devices are portrayed not merely as tools to support participation, but as means of achieving abilities that surpass typical human performance in extraordinary ways.

Such narratives are not neutral. By framing disability primarily through exceptional performance or technological enhancement, media coverage can shape public perceptions and social acceptance of assistive technologies, potentially marginalizing everyday users and obscuring the societal responsibility to provide accessible environments. At the same time, narratives that emphasize spectacle and breakthrough technologies may influence which forms of innovation are perceived as desirable or impactful, indirectly signaling priorities to researchers, funders, and industry partners.

The media often situate the Cybathlon within a transhumanist imaginary, portraying the event as a showcase for “cyborg athletes” and the future of human enhancement. As Barker and Parker [4] and Richard and Andrieu [22] argue, this framing can be problematic. The core significance of the Cybathlon lies not in promoting a transhumanist ideal, but in making visible the diversity of human–technology interactions and challenging normative assumptions about ability. The event provides a platform for “capability hybridization,” in which new forms of embodiment, agency, and participation are explored.

However, dominant media discourses have yet to fully embrace this perspective, often defaulting to narratives of overcoming and spectacle. This points to a broader “zone of friction” in how hybrid performances are represented and understood, and underscores the need for more critical and inclusive media approaches to disability futures [4, 22, 27]. We therefore suggest that future media strategies emphasize the everyday relevance of assistive technologies and their broader societal implications, linking innovation more explicitly to social inclusion rather than exceptional performance alone.

#### Limitations

While the dataset used in this study provides a robust overview of Cybathlon’s media coverage, it may not capture every mention across all platforms nor fully represent all regions. Therefore, the results should be interpreted as a comprehensive, but not exhaustive, mapping of the event’s media impact. Several limitations affected data collection and analysis. Determining whether an article was published exclusively online, in print, or in both formats was often challenging, potentially leading to overlap or underrepresentation in the dataset. Despite the combination of manual and automated search methods, the dataset is not fully inclusive; numerous media articles, particularly on local websites or in print media across different countries, are likely to remain unaccounted for. One of the software tools used, Muck Rack, provided coverage only from November 2019 to December 2024, which may have resulted in missing data, especially around the first Cybathlon in 2016. Additionally, it does not include articles behind paid subscriptions (e.g., The New York Times). Platforms such as Spotify were excluded due to tracking limitations, potentially omitting relevant podcast mentions. Instagram and Facebook were also excluded because comprehensive tracking and data extraction were not feasible.

Furthermore, simply listing the number of articles does not provide any information about how many people were reached or what financial value this represents. Neither the Cybathlon team nor the authors of this article have made an attempt to convert media coverage into monetary terms. However, as there were reports on major television channels, e.g., British Broadcasting Corporation (BBC), Nippon Hōsō Kyōkai (NHK), and online newspapers like The New York Times, it can be assumed that millions of people around the world were informed about the Cybathlon.

These limitations primarily affect the interpretation of dominant media narratives rather than the completeness of coverage, and caution against generalizing media framings to societal attitudes or user perspectives.

### Discussion of scientific impact

#### Cybathlon as a niche

The scientific landscape surrounding Cybathlon can best be characterized as a niche yet emerging research domain. Originating from the ground up, it has developed into a small but dynamic community of researchers, engineers, students, and clinicians dedicated to assistive technologies and inclusive competition. Through its unique format and interdisciplinary approach, Cybathlon provides a platform where novel ideas can be tested, refined, and gradually disseminated. Although its representation in major academic databases remains limited, the presence of Cybathlon-related research indicates growing recognition and impact. This trajectory underscores the importance of sustained knowledge exchange within the Cybathlon community, which serves as a foundation for future innovation in assistive technology research and development.

#### Value of scientific publications and international collaboration

Despite its niche status, Cybathlon has already generated a substantial and diverse body of scientific literature. Nearly 300 identified publications cover a wide range of disciplines, including FES, BCI, EXO, ARM, and LEG, reflecting the event’s role as a platform for cross-disciplinary innovation and knowledge exchange. About one third of these publications explicitly mention the Cybathlon in the title, indicating a high level of direct event-related visibility, while others may still focus primarily on the competition without naming it explicitly in the title, particularly in non-English media or due to editorial naming conventions. The international distribution of publishing authors further demonstrates Cybathlon’s engagement with a global research community, which is essential for sharing best practices and accelerating the development of novel solutions for people with disabilities.

To estimate the number of publications, comparable recurring events can be used as reference points. A quick search in Google Scholar for the period 2014–2025 yields 37,000 results for the term “Olympics,” 16,200 for “Paralympics,” 8,200 for “RoboCup soccer,” and 4,300 for “DARPA robotics challenge,” compared with 1,800 results for “Cybathlon.” The relatively low number of results for the Cybathlon is likely due mainly to its shorter history and the more limited resources available for its organization. In parasport, increased attention is a recent development, with research on equality and inclusion now growing alongside the examination of sporting performance itself. This type of research does not typically accompany purely technical competitions or challenges such as the RoboCup or those created by the DARPA (Defense Advanced Research Projects Agency). These events have primarily targeted university teams and, unlike Cybathlon, DARPA has consistently offered very high prize money [20]. Because the solutions developed for these technical challenges often address markets far larger than rehabilitation robotics, such as autonomous driving, while human involvement is treated as a secondary concern or even as an obstacle in most of them (with the exception of the ANA Avatar XPRIZE competition on teleoperation [9], the current number of publications on the Cybathlon can be considered a strong indicator of its success. And, in contrast to competitions such as the DARPA Challenges or RoboCup, which primarily generate technology-centric knowledge on autonomy and system performance, Cybathlon produces empirical insights into human–technology interaction, user-centered design, and the translation of assistive technologies into clinical and everyday contexts.

To further contextualize the scientific output associated with the Cybathlon, the identified publications can be differentiated by the nature of the knowledge they generate. The literature is dominated by technical studies, reflecting the competition’s strong roots in engineering-driven innovation, with a substantial share consisting of review, survey, or overview articles. In contrast, fewer publications address clinical and therapeutic aspects, usability and user-centered design, or broader societal, ethical, and educational perspectives. While these areas currently represent a smaller proportion of the literature, they are central to the translation of assistive technologies into real-world use and societal adoption.

#### Implifications for future research directions

The observed growth and diversification of Cybathlon-related publications and collaborations suggest that the competition has functioned as a catalyst for interdisciplinary research at the intersection of rehabilitation, assistive robotics, and user-centered design. The bibliometric patterns indicate a shift toward application-driven research questions, increased collaboration between engineering and clinical partners, and a stronger emphasis on real-world usability and benchmarking under functional constraints. These trends point toward future research directions that prioritize participatory design, standardized evaluation protocols, and closer integration of end users into early-stage technology development.

#### Limitations

The publications identified in this study should be considered a broad overview rather than a fully exhaustive inventory, for several reasons. First, some searches were conducted manually, which may have resulted in the omission of publications, particularly those published in languages other than English, where the event may be referenced using alternative terms or non-Latin scripts, such as Japanese, Hindi, Russian, Korean, or Chinese. Second, only publications explicitly mentioning the term “Cybathlon” were included. Works referring to the event using alternative expressions, such as “Cyberolympics” or “Cyborg Olympics,” or those discussing assistive technologies showcased at the Cybathlon without directly naming the event, were not captured in this analysis.

Attributing specific scientific advances causally to the Cybathlon is inherently challenging. The event was not designed as a prospective study with predefined control variables, standardized outcome measures, or systematic surveys of the broader research community. As a result, changes in research trajectories, device development, or clinical methodologies can only be assessed retrospectively and indirectly through publications and reported development pathways. Moreover, the Cybathlon’s influence likely extends beyond participating teams, as many researchers may have been inspired without direct participation, an effect that cannot be captured by participation-based analyses alone.

Consequently, bibliometric indicators should be interpreted as proxies for visibility and scientific engagement rather than as causal evidence of Cybathlon-driven research outcomes.

### Experience of developers

#### Survey reach and representativeness

A notable strength of this survey is the high response rate of 62.3% among all teams that participated since 2016, which is well above the average response rate for online surveys (approximately 44%; [30], despite the fact that the average time taken to complete the entire questionnaire was quite long (55 min). This high level of engagement not only strengthens the reliability of the findings but also reflects the strong sense of community and commitment within the Cybathlon network. The response rate provides a solid foundation for assessing Cybathlon’s impact and can be viewed as a conservative estimate of the event’s broader influence. It is likely that the actual reach of Cybathlon extends even further.

#### Educational impact

Our survey responses suggest that Cybathlon has made a significant contribution to advancing innovation in academia and industry on a global scale. Across Europe and internationally, the themes and disciplines related to Cybathlon have been integrated into curricula at technical universities, universities of applied sciences, and medical faculties. This broad uptake across diverse institutional contexts ensures that future engineers, clinicians, and students from interdisciplinary fields are exposed to the challenges and opportunities associated with assistive technologies. As reported by team managers and members, examples of this impact include:

- Integrating Cybathlon-inspired challenges into university courses on AI, robotics, and biomedical engineering.
- Developing new curricula focused on rehabilitation technologies and movement control.
- Organizing public outreach events and live demonstrations of assistive devices.
- Creating teaching materials and case studies based on Cybathlon experiences.
- Hosting guest lectures and workshops featuring competition participants and experts.

The diversity of educational formats highlights Cybathlon’s capacity to support critical thinking and problem-solving skills - just as it has been described for other robotic challenges and contests [15].

#### Clinical practice and rehabilitation

Our analysis of the survey responses indicates that over one-third of the respondents reported that Cybathlon had a direct impact on the development of new rehabilitation methods and therapies, as well as on fostering collaborations with clinical institutions. The data collected reported progress in the design and control of prosthetic devices, exoskeletons, assistive robotics, and FES systems. Innovations include improvements in device adaptability, usability, and control strategies, such as advanced algorithms for FES and prosthetics, often informed by direct feedback from end-users. The involvement of end-users was certainly encouraged by the Cybathlon rules, which explicitly defined everyday challenges that are most likely to be solved efficiently through interaction between humans and technology. Collaboration with the end-users has already been elaborated as key for successful participation in the Cybathlon competition [17]. The involvement of people with disabilities in the development of (physical) assistive robotics is widespread – however, most developments are only tested on healthy individuals [19]. The Cybathlon addresses this issue, as end users and technology must ‘perform’ at a set time on predefined tasks. Accordingly, several teams highlighted that their involvement in the Cybathlon led to the refinement or consolidation of rehabilitation protocols, both in clinical and home environments. Training for the event prompted the development of new benchmarks and assessment tools, which have been used to structure rehabilitation programs and evaluate user performance in areas such as agility, strength, and balance. In some cases, the team managers and members introduced virtual training systems and novel control methods for assistive devices, further broadening the scope of rehabilitation practice. The competition tasks were also used to assess technological suitability for activities of daily living based on established, observational-based assessments, e.g., via the Assessment of Capacity for Myoelectric Control for arm prostheses [5].

Further developments were noted in the areas of mobility and sensory feedback, including the creation of wearable and non-invasive systems. These advances have aimed to enhance user experience and autonomy, both in clinical environments and at home.

The teams also reported that the Cybathlon provided them with the opportunity to begin and foster collaborations with clinics, hospitals, and rehabilitation centers mostly in Europe, North America, and Asia. These collaborations have resulted in the clinical application and testing of novel devices, as well as the modification of assistive technologies to fit different clinical settings and patient populations.

Overall, the findings suggest that Cybathlon has played a crucial role in supporting clinical innovation on a global scale by facilitating the development, assessment, and implementation of new rehabilitation technologies and methods.

#### Implications for developers and practitioners

For developers and practitioners in robotic neurorehabilitation, the Cybathlon experience highlights several practical lessons. Survey responses and reported development trajectories indicate that early and sustained user involvement, combined with close collaboration between engineers, clinicians, and pilots, facilitates rapid iteration under realistic, task-oriented conditions. The competition setting exposes usability, robustness, and integration challenges that are often not apparent in laboratory-only evaluations. In addition, Cybathlon participation underscores the importance of anticipating regulatory, clinical, and translational constraints early in the development process, as these factors strongly influence whether promising prototypes can progress beyond experimental use.

#### Technology transfer

According to survey responses, Cybathlon participation was reported as a necessary condition for the founding of three companies, while it facilitated (but was not essential to) the founding of five additional companies. Several startups and established companies also participated in multiple editions of the competition. Here, too, a comparison can be made with other competitions: participants in the DARPA Challenges founded various globally active companies (e.g., Waymo, Argo AI, Aurora Innovation), and warehouse automation has also benefited significantly from RoboCup, as Kiva Systems emerged from the four-time winner of one of the leagues [15]. However, the comparison is flawed, as the market for rehabilitation robotics is considered to be significantly smaller. In addition, the 45 non-responding teams may further increase the number of companies whose development was facilitated by the event. Indeed, some companies known to have been founded because of Cybathlon are absent from our list. Moreover, companies not founded as a direct consequence of the Cybathlon have nonetheless benefited from the platform by releasing new product versions, benchmarking their products, using the competition for advertising, or learning from observing other teams [13].

Transitioning from company formation to product development, survey data indicate that 44 products were reported as having been “facilitated” by participation in Cybathlon, and 19 as “newly developed.” These figures, however, require careful interpretation. Many respondents appeared to equate “product” with any newly created device or prototype rather than a commercially available solution. This ambiguity, common in technology-driven fields where the boundary between prototype and market-ready product is often blurred, is underscored by the fact that only 11 teams reported having a product available on the market. This finding highlights the persistent gap between development and successful commercialization.

The transition from a research prototype to a commercial product that can be used in daily life remains a major challenge [13]. As reflected in the responses from many teams, only a minority of teams succeeded in bringing their innovations to the market. According to the answers in our questionnaire, the main obstacles to commercialization are:

- Prototypes often being in early development stages or specifically tailored for the competition, requiring further refinement and testing before they are suitable for everyday use.
- A primary focus on research, innovation, or competition performance rather than commercialization.
- Difficulties in attracting sustained industry interest or protecting intellectual property in a specialized and highly regulated market.
- Limited market size, unclear business cases, or the need for additional development to meet user needs.
- Regulatory and certification requirements, such as compliance with medical device standards, which add substantial complexity, time, and cost.
- Financial constraints and lack of resources, particularly among student teams and research institutes not structured for long-term product development or large-scale commercialization.
- Discontinuity after the event, especially for student-led teams, due to graduation and alternative career paths, which can interrupt further development and technology transfer.
- The inherently slow nature of technology transfer and market insertion, meaning that teams participating only in recent editions (2020 or 2024) often had insufficient time to progress toward commercialization.

In addition, some teams participated with devices that were already commercially available. For these teams, Cybathlon participation did not aim at developing new products but instead served as benchmarking, validation under realistic conditions, and increased visibility, which in turn motivated and informed other participants. In contrast, Cybathlon enabled or supported the success establishment of companies and many others used the event to introduce new product versions. Together, these observations illustrate that the Cybathlon supports commercialization in multiple ways, while also highlighting structural, financial, and temporal constraints that shape translation outcomes.

The mention of economic and financial barriers such as inadequate access to capital, small market size and a highly fragmented healthcare sector is consistent with other surveys in the field of assistive robotics [1]. Even if only a few products ultimately reach the market, it is notable that 63 of the 80 responding team managers and members recognized Cybathlon’s role in facilitating prototype development. This finding underscores that Cybathlon functions not merely as a competition but as a catalyst for innovation and collaboration. By connecting vision with practical application, the event provides momentum for projects to advance toward commercialization. With continued support, Cybathlon has the potential to further expand its impact on people with disabilities worldwide.

Cybathlon appears to serve different stakeholder groups in distinct ways. Academic teams and small enterprises often use the event as a development and validation platform, while established companies may engage more selectively, for example to benchmark technologies, gain visibility, or present product iterations. Based on the available data, no clear longitudinal trend toward increased or decreased participation by specific stakeholder categories can be identified. Moreover, participation levels alone do not capture broader forms of engagement, such as strategic observation, knowledge transfer, or indirect influence on research and development trajectories.

#### Funding sources

The data reveal a clear distinction between traditional funding sources and the use of crowdfunding among Cybathlon participants. A large majority of teams reported receiving financial support for their projects, reflecting the resource-intensive nature of assistive technology development, where expenses for hardware, software, prototyping, and personnel often require backing from public institutions, universities, or private sponsors. This high prevalence suggests that securing funding is not only common but may also be essential for participation in an event such as Cybathlon, which demands substantial investment in research and development. In contrast, only a minority of teams utilized crowdfunding, indicating that such platforms may be less suitable or less familiar in this context. Contributing factors could include the niche nature of the projects, limited public appeal, or insufficient resources to manage campaigns. Overall, while traditional funding is widespread and often necessary, crowdfunding remains an underutilized mechanism for supporting Cybathlon-related initiatives.

#### Public visibility and political organizations

Cybathlon clearly enhances public visibility, providing teams with broader recognition and elevating discourse on assistive technologies. However, engagement with NGOs and other civil society actors remains limited, indicating an opportunity to expand the ecosystem. Strengthening these connections could support longer-term impact by facilitating technology implementation, promoting social inclusion, and advancing accessibility advocacy.

#### Translation into devices and methodological improvements

While causal attribution is not possible, survey responses indicate that Cybathlon participation has translated into concrete, practice-oriented improvements. Teams report refinements in hardware robustness, control strategies, training protocols, and evaluation methods, often driven by the need to perform reliably under competition conditions that resemble daily-life challenges. Several respondents highlighted that the competition accelerated iterative testing with users, shortened feedback loops between engineers and clinicians, and influenced rehabilitation methodologies by emphasizing functional performance over laboratory-only metrics.

#### Limitations

While the survey provides valuable insights into the impact of Cybathlon on participating teams, several limitations should be considered. First, the representativeness and consistency of responses varied. Teams sometimes reported multiple sub-teams as separate entities, while others listed only the main organization, and several teams had multiple respondents, occasionally under slightly different names. This introduces some uncertainty in attributing responses and may result in overrepresentation of certain teams.

Language and distribution also pose limitations. The questionnaire was administered exclusively in English, which may not have been the first language for all participants, potentially affecting comprehension.

The survey’s scope further limits interpretation. It did not explicitly assess whether investments in time, finances, or travel translated into measurable returns, leaving the cost-benefit dimension unexplored. As with any online survey, response bias, including social desirability, recall, recency, and acquiescence biases, may have influenced the results. Finally, while the response rate among managers and team members was relatively high (64%), non-respondents may differ systematically, further constraining generalizability.

Despite these limitations, the survey offers an important snapshot of Cybathlon’s perceived impact on participating teams and provides a foundation for future, more comprehensive studies.

These factors limit the generalizability of the survey findings to the broader research and clinical community, particularly to groups influenced by Cybathlon without direct participation.

### Outlook

Beyond methodological considerations, the Cybathlon initiative itself is expected to evolve substantially in the coming years. Planned developments include hosting future main championships in different countries, introducing new disciplines and competitive formats, and expanding participation by teams, pilots, audiences, and industrial partners. These steps aim to increase international reach, societal visibility, and opportunities for technology transfer and company formation. In this sense, Cybathlon increasingly aspires to function not only as a recurring competition, but as a growing, international movement, comparable in spirit, though not in scale, to established sporting movements, centered on inclusion, innovation, and human–technology interaction.

## Conclusion

The analysis of Cybathlon’s media coverage from 2014 to 2025 reveals both the event’s growing visibility and its evolving role in shaping public discourse around innovative assistive technologies and disability. The data demonstrates that Cybathlon has established a strong and growing presence across multiple media platforms, with significant peaks in coverage corresponding to the main event years. This pattern underscores the event’s capacity to capture public and journalistic interest, particularly as it continues to carve out its own identity within the field of technological innovation.

Over the past decade, Cybathlon has played an increasingly relevant role in promoting innovation of assistive devices within the scientific community. The steady increase of research activities indicates that Cybathlon has inspired a range of scientific work, contributing to developments in device design, clinical practice, and rehabilitation science. The diversity of topics and disciplines represented in the nearly 300 identified publications highlights the event’s capacity to encourage interdisciplinary collaboration and address both technical challenges and user experiences. A recent survey among Cybathlon participants confirmed that the event achieves its conceptual goal of promoting active user involvement in design and development, with 85% of pilots reporting direct participation in the process [13, 17]. This high level of user engagement is likely to contribute positively to the development of appropriate and effective assistive technologies in the long term.

Cybathlon has a significant and multifaceted impact on participating teams, enhancing visibility, credibility, and recognition within the assistive technology community. The event fosters collaboration among researchers, engineers, clinicians, and users, thus facilitating the development, testing, and refinement of prototypes and increasing the likelihood of translation into commercial products. Overall, the Cybathlon has established itself as a structured, recurring platform that connects research, clinical practice, and commercialization, and serves as a sustained driver of innovation in assistive technologies. A decade of Cybathlon experience highlights the importance of early and sustained interdisciplinary collaboration between engineers, clinicians, and end users to ensure relevance beyond laboratory settings. Continuous user integration under realistic conditions can accelerate iteration and improve usability, while regulatory, certification, and commercialization requirements should be anticipated early in development. Finally, sustained support structures beyond competition events, such as follow-on funding and industry partnerships, are critical to translate prototypes into long-term clinical and societal impact.

Future studies could build on this work by applying multilingual and multi-term search strategies, extending analyses to additional social media platforms, and combining bibliometric data with prospective surveys designed to capture longer-term scientific and clinical impacts, particularly as the Cybathlon expands internationally, introduces new disciplines and formats, and grows in societal visibility, industrial engagement, and technology transfer.

## Supplementary Information

Below is the link to the electronic supplementary material.

Supplementary Material 1.

## Data Availability

The detailed responses from the team survey can be requested from the authors.

## References

[1] Aguiar Noury G, Walmsley A, Jones RB, Gaudl SE. The Barriers of the Assistive Robotics Market—What. Inhibits Health Innovation? Sensors. 2021;21(9):3111. 10.3390/s21093111. 33947063 10.3390/s21093111 PMC8125645

[2] Anderson M, Jiang J. (2018). Teens, social media & technology 2018. *Pew research center*, 31(2018), 1673–1689.

[3] Harzing AW. (2007). *Publish or Perish* [Computer software]. https://harzing.com/resources/publish-or-perish

[4] Barker N, Parker H. Hybrid performances in sport: Cybathlon spectatorship for critically imagining technologies for disability futures. Med Humanit. 2024;50(4):657–69. 10.1136/medhum-2024-013031. 10.1136/medhum-2024-013031 PMC11877034 39674592

[5] Capsi-Morales P, Piazza C, Sjoberg L, Catalano MG, Grioli G, Bicchi A, Hermansson LM. Functional assessment of current upper limb prostheses: An integrated clinical and technological perspective. PLoS ONE. 2023;18(8):e0289978. 10.1371/journal.pone.0289978. 37585427 10.1371/journal.pone.0289978 PMC10431634

[6] China Widener J, Arbanas D, Van Dyke C, Arkenberg B, Brooke Auxier. Matheson, &. (2025). 2025 Digital Media Trends: Social platforms are becoming a dominant force in media and entertainment. Deloitte Insights. https://www2.deloitte.com/us/en/insights/industry/technology/digital-media-trends-consumption-habits-survey/2025.html

[7] Greenwood S, Perrin A, Duggan M. Social media update 2016. Pew Res Cent. 2016;11(2):1–18.

[8] Guo M, Wang Y, Yang Q, Li R, Zhao Y, Li C, Zhu M, Cui Y, Jiang X, Sheng S, Li Q, Gao R. Normal Workflow and Key Strategies for Data Cleaning Toward Real-World Data: Viewpoint. Interact J Med Res. 2023;12:e44310. 10.2196/44310. 37733421 10.2196/44310 PMC10557005

[9] Hauser K, Watson E, ‘Nell,’ Bae J, Bankston J, Behnke S, Borgia B, Catalano MG, Dafarra S, Van Erp JBF, Ferris T, Fishel J, Hoffman G, Ivaldi S, Kanehiro F, Kheddar A, Lannuzel G, Morie JF, Naughton P, NGuyen S, Locke D. Analysis and Perspectives on the ANA Avatar XPRIZE Competition. Int J Social Robot. 2025;17(3):473–504. 10.1007/s12369-023-01095-w.

[10] Hover P, Dijk B, Breedveld K, van Eekeren F, Slender H. (2016). *Creating social impact with sport events*. 53.

[11] IBAN. (2025). *List of country codes by alpha-2, alpha-3 code (ISO 3166)*. https://www.iban.com/country-codes

[12] International Telecommunication Union (ITU). (2023). *Measuring digital development—Facts and Figures: Focus on Least Developed Countries*. 29.

[13] Jaeger L, Baptista RDS, Basla C, Capsi-Morales P, Kim YK, Nakajima S, Piazza C, Sommerhalder M, Tonin L, Valle G, Riener R, Sigrist R. How the CYBATHLON Competition Has Advanced Assistive Technologies. Annual Rev Control Rob Auton Syst. 2023;6(1):447–76. 10.1146/annurev-control-071822-095355.

[14] Kotu V, Deshpande B. (2019). Data Exploration. In *Data Science* (pp. 39–64). Elsevier. 10.1016/B978-0-12-814761-0.00003-4

[15] Lima PU, Azevedo C, Serra R. (2025). Robot Contests as a Catalyst for Robotics Science. *Annual Review of Control, Robotics, and Autonomous Systems*, *9*.

[16] Meyer JT. (2022). *A Methodological Framework to Support the User-Centered Design of Wearable Robotic Devices for Rehabilitation* [ETH Zurich]. https://www.research-collection.ethz.ch/handle/20.500.11850/566919

[17] Meyer JT, Weber S, Jäger L, Sigrist R, Gassert R, Lambercy O. A survey on the influence of CYBATHLON on the development and acceptance of advanced assistive technologies. J Neuroeng Rehabil. 2022;19(1):38. 10.1186/s12984-022-01015-5. 35366930 10.1186/s12984-022-01015-5 PMC8976279

[18] Mitra S, Paterson C. Reporting Global While Being Local: Local Sources of News for Distant Audiences. Journalism Stud. 2019;20(12):1671–8. 10.1080/1461670X.2019.1639540.

[19] Nanavati A, Ranganeni V, Cakmak M. Physically Assistive Robots: A Systematic Review of Mobile and Manipulator Robots That Physically Assist People with Disabilities. Annual Rev Control Rob Auton Syst. 2023;7(1):123–47. 10.1146/annurev-control-062823-024352. 10.1146/annurev-control-062823-024352 PMC12978752 41822562

[20] Nardi D, Roberts J, Veloso M, Fletcher L. Robotics Competitions and Challenges. Springer Handbook of Robotic. 2nd ed. Springer; 2016. pp. 1759–88.

[21] Python Software Foundation. (2023). *Python* (Version 3.12.1) [Computer software]. https://www.python.org/

[22] Richard R, Andrieu B. The Cybathlon experience: Beyond transhumanism to capability hybridization. J Philos Sport. 2019;46(1):49–62. 10.1080/00948705.2018.1561297.

[23] Riener R. The Cybathlon promotes the development of assistive technology for people with physical disabilities. J Neuroeng Rehabil. 2016;13(1):49. 10.1186/s12984-016-0157-2. 27246601 10.1186/s12984-016-0157-2 PMC4886429

[24] Riener R, Seward LJ. (2014). Cybathlon 2016. *2014 IEEE International Conference on Systems, Man, and Cybernetics (SMC)*, 2792–2794. 10.1109/SMC.2014.6974351

[25] Roman Widen. Digitalization and Media Consumption: Shaping the Future of Content Engagement. Global Media J. 2024;22(72:456):3.

[26] Tweet Binder. (2025). [Computer software]. Audiense Limited. https://www.tweetbinder.com/

[27] Wolbring G. (2018). Prostheses and Other Equipment: The Issue of the Cyborg Athlete—Interrogating the Media Coverage of the Cybathlon 2016 Event. In I. Brittain & A. Beacom, editors, *The Palgrave Handbook of Paralympic Studies* (pp. 439–459). Palgrave Macmillan UK. 10.1057/978-1-137-47901-3_20

[28] World Health Organization. Global Report on Health Equity for Persons with Disabilities. 1st ed. World Health Organization; 2022.

[29] World Health Organization. (2025, May 23). *Disabilities | WHO | Regional Office for Africa*. https://www.afro.who.int/health-topics/disabilities

[30] Wu M-J, Zhao K, Fils-Aime F. Response rates of online surveys in published research: A meta-analysis. Computers Hum Behav Rep. 2022;7:100206. 10.1016/j.chbr.2022.100206.

