# Correction: A decade of Cybathlon: impact on public visibility, scientific dissemination and technology transfer

**DOI:** 10.1186/s12984-026-02034-2

**Published:** 2026-06-03

**Authors:** Robert Riener, Luca Vassella, Peter Wolf

**Affiliations:** 1 Sensory-Motor Systems Lab, ETH Zurich, Zurich, Switzerland; 2 Spinal Cord Injury Center, University of Zurich, Zurich, Switzerland; 3 Graduate School of Engineering, Tohoku University, Sendai, Japan

**Correction: Journal of NeuroEngineering and**

**Rehabilitation (2026) 23:148**

10.1186/s12984-026-01988-7

There is a duplication of a paragraph under the Conclusion section of this article [1], where the same text appears twice consecutively. As a result, the duplicated paragraph has been deleted.

The following paragraph was the one appearing twice:

“Cybathlon has a significant and multifaceted impact on participating teams, enhancing visibility, credibility, and recognition within the assistive technology community. The event fosters collaboration among researchers, engineers, clinicians, and users, thus facilitating the development, testing, and refinement of prototypes and increasing the likelihood of translation into commercial products.”

The original article has been corrected.
