# Supplementary material for: A decade of Cybathlon: impact on public visibility, scientific dissemination and technology transfer
Source: J Neuroeng Rehabil. 2026 Apr 16;23:148. doi: 10.1186/s12984-026-01988-7 (PMC13135255; doi:10.1186/s12984-026-01988-7)
Supplement: Supplementary file 1 — Supplementary Material 1. [file 12984_2026_1988_MOESM1_ESM.pdf]

CYBATHLON - Survey for Teams

This survey is designed for CYBATHLON **Teams** to share their experiences. We want to learn about your participation, your devices, and how your journey through the CYBATHLON has influenced your products. Your responses will help us understand the impact that the CYBATHLON has on promoting innovation in assistive technologies and their adoption in commercial, educational, therapeutic, and media fields.

Please respond in as much detail as possible. If you have participated in multiple editions of the CYBATHLON across different disciplines, we kindly ask you to complete the questionnaire separately for each discipline. Thank you for your input!

Questionnaire created by Luca Vassella (MSc Student in Health Sciences and Technology) in collaboration with Prof. Dr. Robert Riener and Dr. Peter Wolf, Sensory-Motor Systems Lab, ETH Zürich.

* Required

Consent Form

Study Title: Exploring the Impact of Participation in the CYBATHLON on Advanced Assistive Technologies

Conducting Person: Luca Vassella
Contact Project Team: Luca Vassella
Email: lvassella@student.ethz.ch

ETH Zurich
Sensory-Motor Systems Lab
GLC, Gloriastrasse 37/39, 8092 Zurich

Data Protection Officer ETH Zurich: Tomislav Mitar (General Information tomislav.mitar@sl.ethz.ch)

General Information
We invite you to participate in our research project. Your participation is entirely voluntary, and you can withdraw at any time without giving reasons and without facing any disadvantages. Please read the information below carefully, and feel free to ask the conducting person any questions you may have.

What is Investigated and How?
This study explores the impact of participation in the CYBATHLON on research, education, commercialization, and media coverage. We will collect data through a questionnaire, which is expected to take approximately 20 minutes for team managers and members, while pilots will need about 5 minutes for their section. Your participation will help us understand how the CYBATHLON impacts the field of advanced assistive devices.

Who Can Participate?
Participants must have been part of a team that participated in any CYBATHLON event since 2016. There are no specific exclusion criteria.

Data Privacy
Data will primarily be collected anonymously through a questionnaire administered via Microsoft Forms, using an account provided by ETH Zürich as part of its Microsoft 365 subscription. The questionnaire will not directly request personal information; instead, you will provide responses related to your team affiliation, the edition of the CYBATHLON you participated in, and your role (team manager, team member, or pilot), which may lead to your identification. At the end of the questionnaire, you will also have the option to provide your name and email address if you agree to be contacted for further clarification of your answers, if needed. All data will be securely stored in accordance with ETH Zurich's data protection policies, and only authorized project members will have access to it. Results will be published in a master's thesis, academic journals, and shared with you upon request.

Who Reviewed This Study?
This study has been reviewed and approved by the ETH Zurich Ethics Commission. The members of the ETH Zurich Ethics Committee may inspect the original data for audit and control purposes, subject to strict confidentiality.

Complaints Office
If you have any complaints regarding your participation, you can contact the secretariat of the ETH Zurich Ethics Committee at ethics@sl.ethz.ch or +41 44 632 85 72. More information can be found at ETH Zurich Ethics Committee.

Declaration of Consent By signing below, you confirm that:

- I have read and understood the information about the study. Any questions were answered completely and to my satisfaction.
- I have had sufficient time to decide on my participation and am taking part in the study voluntarily.
- I fulfill the stated conditions for participation and am aware that the stated requirements must be met.
- I agree that the data described above may be collected from me and used as described.
- I know that I can cancel my participation at any time.

1. Please select only one of the following answers: *

- ◯ Yes, I have carefully read and understood all the information and agree to participate voluntarily in the study under these conditions.
- ◯ No, I do not wish to participate in this study under these conditions.

Team and Role Information

2. In which **edition** of the CYBATHLON did your team participate? *

- [ ] 2016
- [ ] 2020
- [ ] 2024

3. Please enter your **team's officially registered name** from the **last edition** of CYBATHLON in which you participated. *

4. Please select the **discipline** in which your team participated during your **last edition** of CYBATHLON. *

- ARM
- BCI
- EXO
- FES
- LEG
- ROB
- VIS
- WHL

5. Who are you? *

- Team Manager
- Team Member
- Pilot

Sections for Pilots Only

6. How often **do you use** the technology you brought to the CYBATHLON in your daily life? *

- Daily
- Couple of times per week
- Once a week
- Once a month
- Never

7. Please rate the following aspects related to your experience with the CYBATHLON and the product you use. *

	Not at all	Slightly	Considerably	Extremely
Impact of training for the CYBATHLON on your mobility and physical well-being	○	○	○	○
Involvement in the development process of the product	○	○	○	○
Product satisfaction	○	○	○	○

Research & Education

8. How much has your team's journey through CYBATHLON **contributed** to your research and **facilitated** the publication of articles or papers cited in journals, conferences, seminars, webinars, workshops, or similar platforms? *

- ◯ It happened mainly because of CYBATHLON.
- ◯ CYBATHLON accelerated or facilitated the process.
- ◯ It would have happened anyway, regardless of CYBATHLON.
- ◯ We didn't do any research, nor did we release any publications.

9. Based on your previous answer regarding publications, please list the journals, conferences, societies, etc., where you have published. Additionally, please include any conference talks, seminars, webinars, workshops, etc., where you have showcased your research. *

10. Are you **planning** to release any upcoming **publications** related to your research or **present your findings** at specific conferences or events in the future? *

- ◯ Yes
- ◯ No

11. Based on your previous answer regarding upcoming publications and presenting your findings in the future, could you share more details about it? Please mention the publications you plan to release and the conferences or events you intend to attend to showcase your research. *

12. How much has your team's journey through CYBATHLON contributed to the **development of new educational materials**, **curricula**, **lectures** or **courses**? *

- ◯ It happened mainly because of CYBATHLON.
- ◯ CYBATHLON accelerated or facilitated the process.
- ◯ It would have happened anyway, regardless of CYBATHLON.
- ◯ We did not develop any new educational materials, curricula, lectures, or courses.

13. Based on your previous answer regarding developing new educational materials, please list the new educational material, curricula, lectures or courses your team has developed and specify where they are taking place. *

14. Are you **planning** on developing **new educational materials**, **curricula**, **lectures**, or courses in the **future**? *

- Yes
- No

15. Based on your previous answer regarding future developing of new educational materials, could you share more details about it? Please specify what you plan to develop and any related information. *

Clinical Work

16. How much has your team's journey through CYBATHLON contributed to **developing new rehabilitation methods** or **therapies**, or **consolidating existing ones**? *

- ◯ It happened mainly because of CYBATHLON.
- ◯ CYBATHLON accelerated or facilitated the process.
- ◯ It would have happened anyway, regardless of CYBATHLON.
- ◯ We didn't develop any new rehabilitation methods or therapies, nor did we contribute to the consolidation of existing therapies.

17. Based on your previous answer on rehabilitations methods and therapies, please describe the new rehabilitation methods or therapies your team developed, or how you contributed to consolidating existing ones *

18. Do you plan on **contributing** to the development of new rehabilitation methods/therapies, or consolidating existing ones in the **future**? *

- ◯ Yes
- ◯ No

19. Based on your previous answer regarding future development of therapies, please share more details about it. *

20. How much has your team's journey through CYBATHLON facilitated **collaboration with clinics**, **therapy centers**, or **hospitals**? *

- ◯ It happened mainly because of CYBATHLON.
- ◯ CYBATHLON accelerated or facilitated the process.
- ◯ It would have happened anyway, regardless of CYBATHLON.
- ◯ We didn't collaborate with any clinics, therapy centers, or hospitals.

21. Based on your previous answer regarding collaborations, please provide the names and locations of the clinics, therapy centers, or hospitals you have collaborated with. *

22. Are you planning to **collaborate** with **clinics**, **therapy centers**, or **hospitals** in the **future**? *

- Yes
- No

23. Based on your previous answer regarding future collaborations, please share more details about it. *

Technology Transfer

24. How much has your team's journey through CYBATHLON contributed to the launch of any **startups** or **spin-offs**? Or was your team **already part** of an **established company** before joining the CYBATHLON? *

- ◯ It happened mainly because of CYBATHLON.
- ◯ CYBATHLON accelerated or facilitated the process.
- ◯ It would have happened anyway, regardless of CYBATHLON.
- ◯ We were already part of an established company before joining the CYBATHLON.
- ◯ We have not launched any startups or spinoffs, nor are we affiliated with an established company.

25. Please provide the name of the startup or spin-off (or related entity) that your team has launched. *

26. Please provide the name of the company your team is part of. *

27. Are you considering **starting** any new startups or spin-offs in the **future** that are related to the technology developed for the CYBATHLON? *

- ◯ Yes
- ◯ No

28. Which of the following statements best describes the impact of joining the CYBATHLON on the **development of the product** you brought to the event? *

- ◯ Joining the CYBATHLON led to the creation of a completely new product.
- ◯ Joining the CYBATHLON accelerated or improved the development of the product already in progress.
- ◯ The product would have been developed in the same way even without joining the CYBATHLON.

29. How much has your team's journey through CYBATHLON contributed to the **launch of any new products** or **patents**, or **starting** any **campaigns**? *

- ◯ It happened mainly because of CYBATHLON.
- ◯ CYBATHLON accelerated or facilitated the process.
- ◯ It would have happened anyway, regardless of CYBATHLON.
- ◯ We didn't launch any new products or patents, nor did we start any campaigns.

30. Based on your previous answer regarding launching products or patents, please share any additional details you can provide at this time. *

31. Do you **plan to launch** any new products or patents in the future that are related to the technology developed for the CYBATHLON? *

- ◯ Products
- ◯ Patents
- ◯ Products and Patents
- ◯ We don't plan to launch anything

32. Based on your previous answer regarding the launch of future products and patents, please share any additional details you can provide at this time. *

33. Is the device you brought to the competition **currently available as a product** on the market? *

- ◯ Yes
- ◯ No

34. Do you think participating in the CYBATHLON **has affected** or **will affect** your **sales**? *

	Not at all	Slightly	Considerably	Extremely
Impact on sales	◯	◯	◯	◯

35. If you believe that the CYBATHLON has impacted or will impact your sales, please provide a brief explanation on how.

36. Approximately how many products do you sell annually?

Please provide an approximate number.

37. What was the **status of the product** your team brought to the CYBATHLON? *

- Prototype
- New version (update) of existing model
- Other

38. Have you **sold one or more units** of your product to a hospital, private clinic, individual client, or similar entity? *

- Yes
- No

39. Based on your previous answer regarding selling your product, please share any additional details you can provide. *

40. Do you foresee a point when this product/technology will **officially reach the market**? *

- Yes
- No

41. Approximately in **which year** do you expect your final product to be available on the market? *

Please enter a year.

42. What **reasons** or **obstacles** are preventing your product from reaching the market? *

43. Did you draw any **inspiration** from existing products, other teams/labs, or similar sources when developing your product? *

- Yes
- No

44. You selected "Yes" in the previous question regarding drawing inspiration. From which product did you draw inspiration? *

Please provide the **name** of the product and its developer.

Fundings & Crowfunding

45. Did your team receive any **funding** for the development of the product you brought to the CYBATHLON? *

- ◯ Yes
- ◯ No

46. You selected "Yes" in the previous question regarding fundings. Please select the source of the funding. *

- ☐ Sponsorship from an organisation
- ☐ Collaboration with academic institutions
- ☐ Research and development area of a company
- ☐ Research funding from national organisation
- ☐ Grand/Awards for research
- ☐ Other

47. During your journey to CYBATHLON, did your team engage in any **crowdfunding campaigns** for your product? *

- ◯ Yes
- ◯ No

48. You selected "Yes" in the question regarding crowdfunding. Please provide a brief description of the crowdfunding campaign you were engaged in (including details such as the product, timing, etc.). *

Media & Political Organisations

49. How much has your team's journey through CYBATHLON contributed to **being featured** or **invited** to platforms such as TV, radio, podcasts, newspapers, conference talks, fairs/exhibitions, internet articles, social media, or any other platforms? *

- ◯ It happened mainly because of CYBATHLON.
- ◯ CYBATHLON accelerated or facilitated the process.
- ◯ It would have happened anyway, regardless of CYBATHLON.
- ◯ We haven't been featured, nor did we receive any invitations.

50. Based on your previous answer regarding being featured or invited, can you please select all that apply? *

- ☐ TV
- ☐ Radio
- ☐ Podcasts
- ☐ Newspapers
- ☐ Conference talks
- ☐ Fairs/Exhibitions
- ☐ Internet articles (webpages)
- ☐ Social Media
- ☐ Other

51. Please specify where and with whom you discussed your product (e.g., the name of the television station, podcast, conference, social media). *

52. How much has your team's journey through CYBATHLON contributed to **gaining popularity** on **social media** (Instagram, Twitter, LinkedIn, etc.)? *

- ◯ It happened mainly because of CYBATHLON.
- ◯ CYBATHLON accelerated or facilitated the process.
- ◯ It would have happened anyway, regardless of CYBATHLON.
- ◯ We didn't gain any popularity on social media.

53. How much has your team's journey through CYBATHLON facilitated engagement with **political organisations** or **non-governmental organisations** (**NGOs**)? *

- ◯ It happened mainly because of CYBATHLON.
- ◯ CYBATHLON accelerated or facilitated the process.
- ◯ It would have happened anyway, regardless of CYBATHLON.
- ◯ We didn't engage with any political organisations or NGOs.

54. Based on your previous answer regarding engaging with political organisations or NGOs, please provide a brief description of your team's engagement. *

55. Do you plan to engage with any **political organisations** or **non-governmental organisations** (**NGOs**) in the future? *

- ◯ Yes
- ◯ No

56. Based on your previous answer regarding future engagement with political organisations and NGOs, please share more details about it. *

Concluding Questions

57. Impacts of CYBATHLON *

	Not at all	Slightly	Considerably	Extremely
Impact on technology transfer	○	○	○	○
Impact on teams' reputation	○	○	○	○

58. What aspects make participating in the CYBATHLON worth the journey for you and your team? *

Please select at most 3 options.

- ☐ Winning races
- ☐ Feedback on product/technology
- ☐ Team recognition
- ☐ Increasing brand awareness
- ☐ Media coverage
- ☐ Networking or partnership opportunities
- ☐ Attracting funding opportunities
- ☐ Boost sales or commercial interest in the product/techonolgy
- ☐ Launch of product/techonolgy
- ☐ Gain of experience
- ☐ Community engagement
- ☐ Team commitment
- ☐ Students involvement

Feedback

59. Would you be ready to be contacted to further clarify your answers on the topic if needed? *

- ◯ Yes
- ◯ No

60. Please write your name. *

61. Please share an email address where you can be contacted. *

62. If you have any feedback, comments, or additional observations regarding the questionnaire, please feel free to share them here.

This content is neither created nor endorsed by Microsoft. The data you submit will be sent to the form owner.

Microsoft Forms
